# Actin-Myosin Interaction: Structure, Function and Drug Discovery

**DOI:** 10.3390/ijms19092628

**Published:** 2018-09-05

**Authors:** Piyali Guhathakurta, Ewa Prochniewicz, David D. Thomas

**Affiliations:** Department of Biochemistry, Biophysics and Molecular Biology, University of Minnesota, Minneapolis, MN 55455, USA; pgt@ddt.umn.edu (P.G.); ewa@ddt.umn.edu (E.P.)

**Keywords:** actin, myosin, ATP, fluorescence, FRET, drug discovery, heart failure

## Abstract

Actin-myosin interactions play crucial roles in the generation of cellular force and movement. The molecular mechanism involves structural transitions at the interface between actin and myosin’s catalytic domain, and within myosin’s light chain domain, which contains binding sites for essential (ELC) and regulatory light chains (RLC). High-resolution crystal structures of isolated actin and myosin, along with cryo-electron micrographs of actin-myosin complexes, have been used to construct detailed structural models for actin-myosin interactions. However, these methods are limited by disorder, particularly within the light chain domain, and they do not capture the dynamics within this complex under physiological conditions in solution. Here we highlight the contributions of site-directed fluorescent probes and time-resolved fluorescence resonance energy transfer (TR-FRET) in understanding the structural dynamics of the actin-myosin complex in solution. A donor fluorescent probe on actin and an acceptor fluorescent probe on myosin, together with high performance TR-FRET, directly resolves structural states in the bound actin-myosin complex during its interaction with adenosine triphosphate (ATP). Results from these studies have profound implications for understanding the contractile function of actomyosin and establish the feasibility for the discovery of allosteric modulators of the actin-myosin interaction, with the ultimate goal of developing therapies for muscle disorders.

## 1. Introduction

A principal challenge in the biophysics of motility is to understand the molecular mechanism by which motor proteins such as myosin convert chemical energy to mechanical work through cyclic interaction with actin filaments. During the actin-myosin ATPase cycle, myosin alters its affinity for actin in a nucleotide-dependent manner from weak (W) actin-binding states [with adenosine triphosphate (ATP) or adenosine diphosphate.phosphate (ADP.P) bound to the myosin active site] to strong (S) actin-binding states (with ADP or no nucleotide, i.e., rigor), resulting in the generation of force. It is extremely challenging to obtain direct information about the structural dynamics of the entire actin-myosin complex, because both actin and myosin undergo changes in structure and dynamics during transitions from the W to S states [1,2,3]. S states are relatively stable and easy to study, but the W state is less accessible to study, due to its transient and structurally dynamic nature.

Recent crystallographic structures of various isoforms of myosin [4] and cryo-electron micrographs of actin-myosin complexes [5,6] have provided important insights about the structure and function of the entire complex. However, these are snapshots of a single structural state. Time-resolved fluorescence resonance energy transfer (TR-FRET) is a high-precision technique that has a key advantage. By measurement of fluorescence lifetimes with subnanosecond resolution within a donor-acceptor complex, TR-FRET can resolve and measure intermolecular distances within a bound protein complex without the interference of unbound proteins. This is especially important for measurements in the presence of ATP, when myosin has lower affinity for actin, so the bound actin-myosin complex represents only a fraction of the total protein population. The direct detection of structural changes within the actin-myosin complex by FRET requires introduction of fluorescent probes at specific sites on both actin and myosin. Skeletal muscle actin has a native cysteine residue, Cys 374 [7], which has been used frequently for introduction of spectroscopic probes. Similarly, one reactive site in the catalytic domain of skeletal muscle myosin, Cys 707, has been utilized extensively as a site for spectroscopic probes in previous studies of structural transitions in myosin [8,9]. Site-directed mutagenesis has been used to introduce other Cys labeling sites or genetically encoded fluorescent proteins [10,11,12]. However, both actin and myosin heavy chains are difficult to express genetically, so the choice of labeling sites for spectroscopic probes is also limited. Therefore, myosin light chains or nucleotide analogs [13] are often used.

Skeletal and cardiac muscle myosin each have a catalytic domain (CD) containing the binding sites of actin and nucleotide, and have a light chain domain (LCD) containing the binding sites for essential (ELC) and regulatory (RLC) light chains. Light chains are important regulators of actin-myosin interactions. In mammalian muscle, RLC modulates the interaction of the myosin head with actin through phosphorylation of residues near the N-terminus [14] while the removal of ELC from myosin results in a loss of actin filament motility [15] and a reduction in force [16]. These light chains can be genetically expressed and modified, e.g., with spectroscopic probes, and exchanged for endogenous light chains. RLC is located far from actin in the actin-myosin complex and is not a prime choice for FRET probes to study actin-myosin interactions, whereas ELC is closer to actin, at an optimal distance for FRET. Fast skeletal muscle has two ELC isoforms, A1 and A2. The longer A1 isoform contains an additional 40–45 amino acids at the N-terminal region (N-terminal extension) [17]. Previous NMR and cross-linking studies indicated that the N-terminal region of A1 is close to actin [18,19]. In contrast, slow skeletal and cardiac muscle contain only the longer A1 isoform [20]. In cardiac muscle, both ventricular and atrial ELC isoforms are A1 and have N-terminal extensions of approximately the same length as in skeletal A1. Both cardiac A1s are important regulators of cardiac contractility [21]. Smooth muscle and nonmuscle myosins have only the short (A2) isoform [22,23]. Myosins show functional differences due to A1/A2 only in the presence of actin, resulting in much higher catalytic efficiency for A1 [24] and higher in vitro actin motility for A2 [25].

This review describes the recent development, in this laboratory, of fluorescent biosensors that directly detect structural transitions within the actin-myosin complex [26,27,28]. Using a fluorescent donor probe on actin and an acceptor probe on myosin’s ELC, together with unique high-performance TR-FRET instrumentation [29], this work has provided direct insight into the ATP-driven structural transitions within the actin-myosin complex, as influenced by isoforms of ELC [26] and cardiomyopathy mutations in the ELC [27]. Finally, this work enabled the development of a high-throughput fluorescence assay with the goal of finding small molecules that modify the actin-myosin interaction for therapeutic purposes [28]. The latter work was recently featured on the cover of the *Journal of Biological Chemistry.*

## 2. Skeletal Muscle Actin-Myosin Structural Transition Depends on the Myosin ELC Isoform

**Figure 1:**
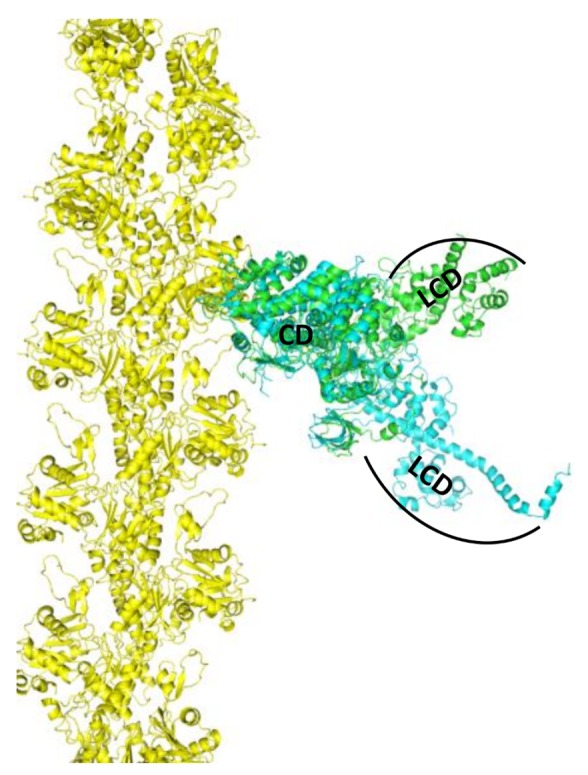
Model, based on superposition of catalytic domain (CD) of myosin crystal structures, for the strong-binding actin-myosin complex (actin yellow, myosin blue, light-chain domain (LCD) down, 2MYS [30]) and weak-binding complex (actin yellow, myosin green, LCD up, 1BR2 [31]). LCD (lever arm) rotation from up to down generates force in the actin-attached power stroke.

The principal goal of muscle structural biophysics is to understand at the atomic level how actin and myosin generate force and motion in contracting muscle. This clearly requires analysis of both proteins in the complex. The current era of muscle structural biophysics really began in the early 1990s, when the high-resolution crystal structures of monomeric actin [2] and myosin S1 [2] were determined. Myosin structure of nucleotide-free chicken skeletal myosin [30] showed that the LCD was oriented “down” with respect to the actin binding interface, and the structure of nucleotide-bound smooth muscle myosin [31] showed that the LCD oriented “up”, as shown in Figure 1. A comparison of these two structures suggested that myosin generates force in the actin-attached power stroke by rotation of the LCD (from up to down), and that this is reversed in the actin-detached recovery stroke. Recent structural insights on the power stroke have come from a high-resolution crystal structure of myosin VI in a previously unseen state [32] and also from a cryo-EM structure of the actin-myosin-MgADP (MagnesiumADP) complex [5]. However, a direct crystal structure of actin-bound S1 is still unavailable, and some of the key proposed structural transitions involve dynamically disordered states, which cannot be defined by static crystal structures.

**Figure 2:**
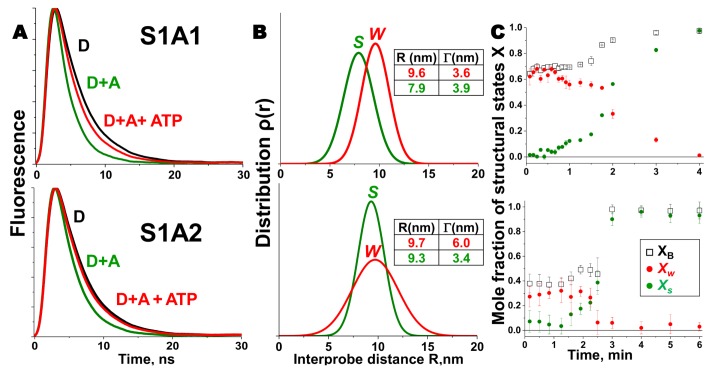
Structural states of the skeletal muscle actin-myosin complex detected by TR-FRET. (**A**) Fluorescence decay of 1 µM AF568 actin (D, black) with 5 µM AF647 labeled S1A1 and 10 µM S1A2 respectively, in the absence (D + A, green) and presence of saturating ATP (D + A + ATP, red), acquired during the steady state. Faster decay in the presence of acceptor indicates FRET. (**B**) Interprobe distance distribution (best fit to a Gaussian function) corresponding to the bound acto-S1 complex, determined from data in (**A**) for *S* (green) and *W* (red) complexes. (**C**) Time dependence of FRET-detected mole fractions of the structural states in B, after addition of ATP (at time 0) to a mixture of donor-labeled actin and acceptor-labeled myosin S1. X_B_ (□, black) is the fraction of donor that has bound acceptor. X_B_ = *X_W_* + *X_S_*, where *X_W_* (red) and *X_s_* (green) are the mole fractions of W and S complexes. Adapted from Guhathakurta, P.; Prochniewicz, E.; Thomas, D.D. Amplitude of the actomyosin power stroke depends strongly on the isoform of the myosin essential light chain. *Proc*. *Natl*. *Acad*. *Sci*. *USA* **2015**, *112*, 4660–4665 [26].

Our TR-FRET study on the actin-myosin complex from skeletal muscle addressed this question directly [26]. We showed that the structural transition in the presence of ATP was different for myosin containing A1 and A2 isoforms of the ELC, which differ by a 45-residue N-terminal extension on A1. Skeletal muscle A1 and A2 were expressed genetically with a single cysteine residue at the C-terminus (C180 in A1 and C136 in A2) of ELC and labeled with an acceptor probe Alexa 647. These ELCs were then exchanged separately onto skeletal muscle S1 (single-headed chymotryptic fragment of the two-headed myosin) to generate homogeneous preparation of S1A1 and S1A2. Actin was labeled at C374 with a donor probe, Alexa 568. TR-FRET measurements on this system, separately for actin-S1A1 and actin-S1A2, showed that the extent of W–S transition in S1A1 was greater than in S1A2, as seen in Figure 2. TR-FRET showed not only that the two isoforms differ in the mean interprobe distance (shorter in rigor for A1 than for A2), but also that there was more disorder in the weakly-bound actin-S1A2 complex, suggesting a tethering role for the N-terminal extension of A1 in the actin-myosin complex, shown in Figure 2. Thus, this biosensor was able to resolve the important structural difference between two actomyosin complexes containing different isoforms of ELC. The application of this sensor can also be extended to study the interaction of actin with non-muscle/smooth-muscle myosin, which contains A2. Since non-muscle myosin is involved in several physiological processes, use of this biosensor will be useful to obtain important information about fundamental biological processes. The tethering role of the N-terminus of A1 was further supported by an additional FRET experiment in which a single cysteine was incorporated at the very N-terminus (C16) of A1. Actin was labeled again at C374 with a different donor probe, 1-5 IAEDANS, and C16 of ELC was labeled with an acceptor, DABCYL Plus C2 maleimide. The measured distance between these two sites was short enough to support the proximity of the N-terminal extension of A1 to actin. The conventional model of LCD rotation during the myosin power stroke, based on crystal structures, did not predict the observed effect of the ELC isoform on LCD movement. Subsequent EM and crystallographic studies on smooth muscle [31], scallop [33], or nonmuscle myosins [34] with short, A2-like ELC, also failed to provide direct information on the role of the N-terminal extension in the power stroke. A detailed modeling study [26] with the three isoforms of myosin showed that the FRET distances observed in the weakly bound skeletal actomyosin complex were consistent with an 18° rotation (detected by electron paramagnetic resonance in a muscle fiber) of the LCD in skeletal muscle. All myosins go through the actomyosin ATPase cycle, but the equilibria and kinetics within the cycle depend on the specific isoform of myosin. The isoform-specific transmission of nucleotide-induced structural changes from the CD to the LCD, which is the essence of the power stroke, is probably necessary to accommodate diverse functional roles of myosins in muscle and nonmuscle cells.

## 3. Cardiac Actin-Myosin Structural Transition Is Affected by a Disease-Causing Mutation

Cardiovascular disorders are the leading cause of morbidity and mortality in the developed world, and hypertrophic cardiomyopathy (HCM) is among the most frequently occurring inherited cardiac disorders. HCM is caused by mutations in the genes encoding the fundamental force-generating machinery of the cardiac muscle. HCM results from mutations in 11 or more different sarcomeric genes [35] and is characterized by left ventricular hypertrophy, cardiomyocyte disarray, and myocardial fibrosis. The clinical manifestations of the disease are quite variable [36], and the wide spectrum of functional perturbations induced by the different HCM mutations suggests that many different pathways lead to the HCM phenotype [35], so it is difficult to establish a prognosis based on the mutation [37]. Although a number of these mutations have been studied using a variety of approaches, there is no clear consensus as to the mechanism(s) by which these mutations give rise to the disease state. A proposed mechanism of HCM mutations is an increase in the number and/or unitary force of force-generating actin-attached myosin heads, producing a hyper-contractile heart [38,39,40]. This is equivalent to the fraction of myosin heads in the strong-binding S state during the steady state of the ATPase cycle, also known as the duty ratio (DR). The DR is typically calculated quite indirectly from several separate measurements, such as actin sliding velocity vs length, optical trap displacement, co-sedimentation assays, and/or the actin dependence of myosin ATPase activity.

**Figure 3:**
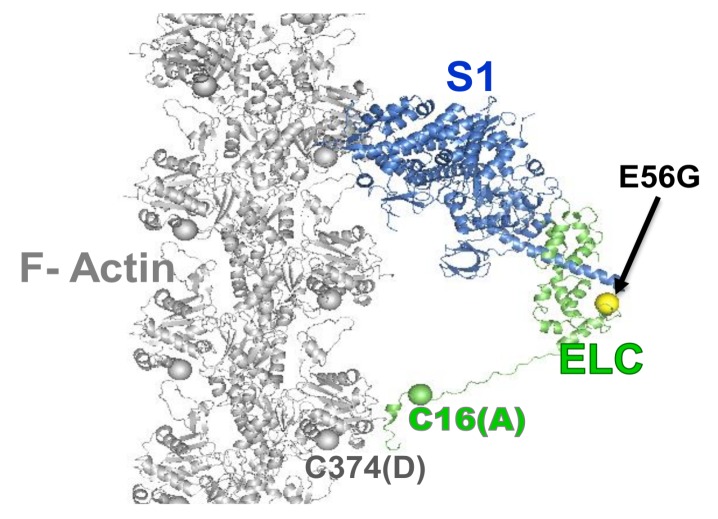
Model of actin-myosin complex with skeletal myosin S1 strongly bound to actin. Spheres show the donor (D) and acceptor (A) labeling sites on actin (C374, grey), on myosin (C16, green), and the HCM mutation (E56G, yellow). The C374(actin)-C16(ELC) FRET sensor was designed to determine the effect of the E56G HCM mutation in hVELC on actomyosin function. Adapted from [27], Guhathakurta, P.; Prochniewicz, E.; Roopnarine, O.; Rohde, J.A.; Thomas, D.D. A Cardiomyopathy Mutation in the Myosin Essential Light Chain Alters Actomyosin Structure. *Biophys*. *J*. **2017**, *113*, 91–100, with permission from Elsevier.

**Figure 4:**
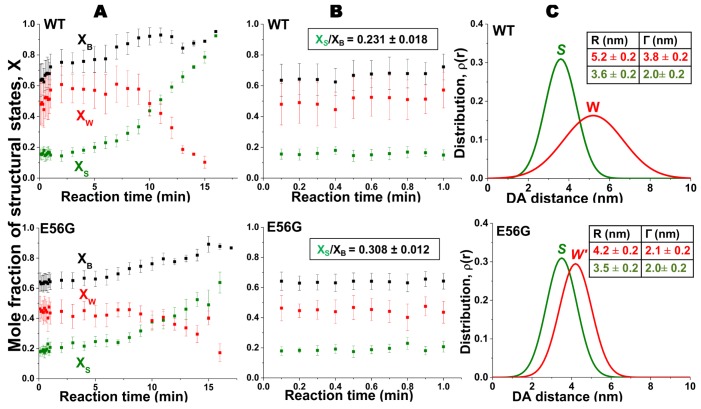
Structural states of actomyosin complex affected by a cardiomyopathy mutation as detected by TR-FRET. (**A**) Time dependence of FRET-detected mole fractions (X) of structural states after addition of ATP to a mixture of donor-labeled actin and acceptor-labeled myosin S1 (WT, top and E56G, bottom). X_B_ (■, black) is the fraction of donor that has bound acceptor. X_B_ = X_W_ + X_S_, where X_W_ (red) and X_S_ (green) are the mole fractions of W and S complexes. (**B**) Time dependence of mole fractions (expanded view) in the first minute after addition of ATP, demonstrating a true steady state. Steady-state duty ratio is shown in the box. (**C**) Interprobe distance (R) and distribution (Γ) (best fit to a Gaussian function) corresponding to bound acto-S1 complex determined from S (green) and W (red) complexes (WT, top and E56G, bottom). While the structure (distance distribution) of the S complex is not significantly affected by the mutation, the W complex is shifted to a shorter distance and a narrower width, so it is designated W′. Each curve is normalized to the unit area, which is independent of the mole fraction X_S_ or X_W_. Thus the distribution of actin-bound distances R at a given time after mixing is given by a linear combination ρ(R) = (X_S_/X_B_)ρ_S_(R) + (X_W_/X_B_)ρ_W_(R). Adapted from [27], Guhathakurta, P.; Prochniewicz, E.; Roopnarine, O.; Rohde, J.A.; Thomas, D.D. A Cardiomyopathy Mutation in the Myosin Essential Light Chain Alters Actomyosin Structure. *Biophys*. *J*. **2017**, *113*, 91–100, with permission from Elsevier.

We extended the use of TR-FRET biosensors to identify the number of interacting actin-bound myosin heads during the ATPase cycle in cardiac myosin. We focused on a mutation in human ventricular (hVELC), E56G, which causes HCM [41]. We hypothesized that this mutation, located near the interface of heavy and light chains, seen in Figure 3, affects cardiac function by altering the structural states of the actin-myosin complex during the ATPase cycle. We expressed a single-cysteine (C16) construct of hVELC without (here designated WT) and with the E56G mutation (here designated E56G). We labeled C16 of hVELC with a non-fluorescent acceptor probe (DABCYL) for both WT and E56G. We then exchanged the acceptor labeled ELCs with the native endogenous ELC in bovine cardiac myosin subfragment 1 (cardiac S1), and made homogenous preparations of WT and E56G cardiac S1. We labeled actin with a fluorescent donor probe (1-5 IAEDANS) at C374. We found that the observed mutation-dependent differences is only significant in the presence of ATP. TR-FRET was used to quantitate the mole fractions, shown in Figure 4A, and structural properties, shown in Figure 4C, of the W and S states. From the mole fractions, we determined the duty ratio (DR), defined as X_S_/X_B_, the fraction of actin-bound heads in the S structural stat; the E56G mutation increased DR by 33% (Figure 4B). The DR is typically calculated from several separate measurements, and is quite indirect [16,40]. However, TR-FRET directly detects and resolves the populations of these structural states of actin-bound myosin heads with high precision in a single measurement. In a recent study, β-cardiac myosin data was analyzed indirectly [42], estimating a duty ratio of 0.20 under our conditions, in excellent agreement with our finding of 0.23, seen in Figure 4. The direct detection of TR-FRET in the bound actin-myosin complex also provides information about the structural properties of the resolved W and S states. In the presence of ATP, there was no effect on FRET for the S complex, but the W complex was clearly affected. The mean distance R_w_ between actin and C16 was decreased to 4.2 ± 0.2 nm in E56G, compared to 5.2 ± 0.2 nm in WT (Figure 4C, red). In WT, the W complex is nearly twice as disordered as the S complex, but this difference is negligible for the E56G mutation, shown in red in Figure 4C. Thus TR-FRET was also able to quantitate the mutation-related structural disorder in the W complex: In the presence of the E56G mutation, the structure of the weak complex is intermediate between those of the W and S complexes of WT cardiac myosin. The increase in the fraction X_S_ of actin-bound myosin molecules that are in the S state causes an increase in the duty ratio for E56G and is consistent with greater force production, as observed in the hypercontractile HCM phenotype [43]. Similarly, the shift of the W structural state in the direction of the S state is consistent with insufficient relaxation (diastole), which has also been proposed for some HCM phenotypes [44]. Thus, this TR-FRET biosensor is a valuable tool for providing direct insight into the subtle structural effects caused directly by a disease-causing HCM mutation. 

## 4. Application of TR-FRET for Drug Discovery

Just as a single missense mutation can alter the structure of the protein of interest and perturb its function, the binding of a small molecule to that protein can do the same, and this is the basis of structure-based drug discovery. This work typically involves the screening of a “library” containing thousands, even millions, of drug-like small molecules by high-throughput screening (HTS). To be truly effective, the structure-based screening method must achieve both high throughput and high precision. At first glance, FRET seems to be an ideal choice for this application, since there are commercially available fluorescence plate readers that can read hundreds of samples per minute. However, these readers typically measure fluorescence intensity, which produces low precision (errors approximately 10%), insufficient for most HTS applications involving FRET. TR-FRET (measured by decreased fluorescence lifetime) offers high precision (errors less than 1%), but commercial plate readers do not offer high throughput. We have recently solved this problem by developing the first high-throughput fluorescence lifetime plate-reader (FLTPR), which achieves both high throughput (hundred of samples per minute) and high precision [12,29,45,46].

**Figure 5:**
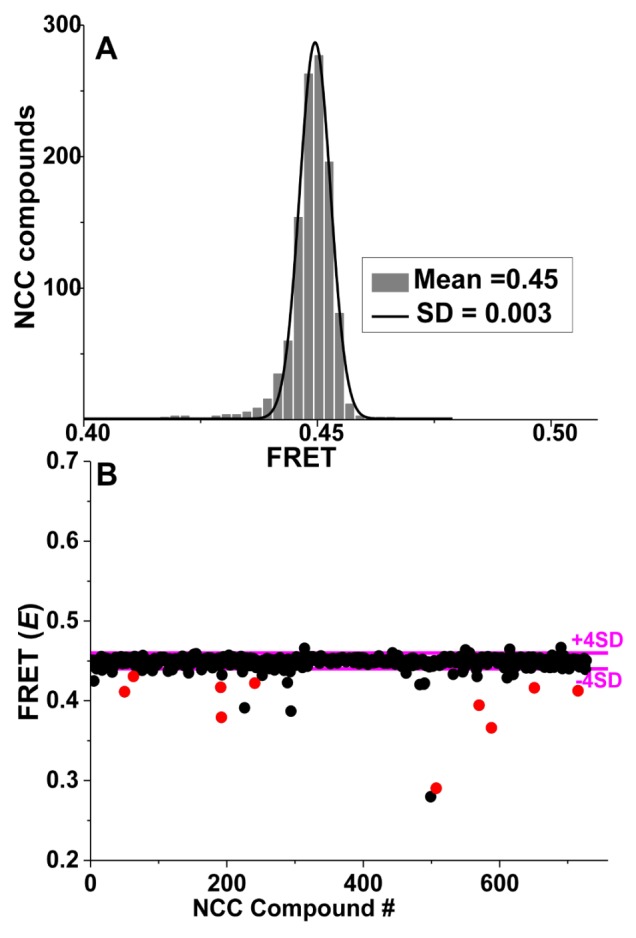
Time-resolved fluorescence resonance energy transfer (TR-FRET) based HTS of National clinical collection (NCC) library for compounds that modulate actin-ANT FRET. (**A**) Histogram plots of all NCC library compounds after removal of fluorescent compounds show an average FRET efficiency of 0.45 ± 0.003. (**B**) FRET from a representative NCC screen with “Hit” threshold (>4 SD of mean) indicated by magenta lines. Reproducible “Hits” from triplicate screens are shown in red. Adapted from Guhathakurta, P.; Prochniewicz, E.; Grant, B.D.; Peterson, K.C.; Thomas, D.D. High-throughput screen, using time-resolved FRET, yields actin-binding compounds that modulate actin-myosin structure and function. *J*. *Biol*. *Chem*. **2018**, *293*, 12288–12298 [28].

**Figure 6:**
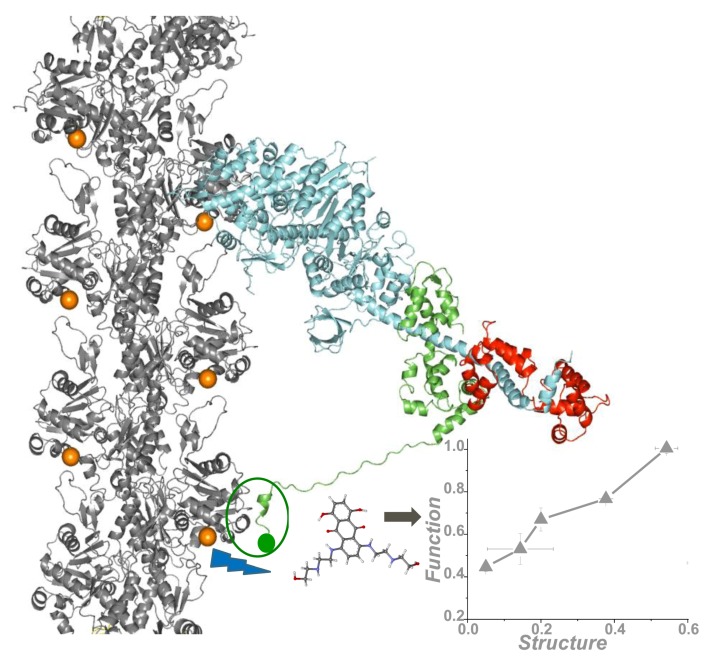
High-throughput fluorescence resonance energy transfer (FRET) from actin labeled at the C-terminus (orange spheres) to a peptide derived from the N terminus (green sphere) of the myosin essential light chain, detects actin-binding compounds that change actomyosin structure and function. Adapted from the cover of Guhathakurta, P.; Prochniewicz, E.; Grant, B.D.; Peterson, K.C.; Thomas, D.D. High-throughput screen, using time-resolved FRET, yields actin-binding compounds that modulate actin-myosin structure and function. *J*. *Biol*. *Chem*. **2018**, *293*, 12288–12298 [28].

Small-molecule effectors designed to modulate actin-myosin function for the treatment of disease are showing promise in preclinical and clinical trials [47,48,49]. However, these compounds were developed via a slow process using low-throughput functional assays. Here we review our recent work using TR-FRET in a structure-based HTS study, offering both high throughput and high precision, for small molecules that perturb the actin-myosin interaction. While our previous TR-FRET studies [26,27] used donor-labeled actin and fluorescent labeled myosin S1, we found that the large-scale production of labeled myosin S1, as needed for HTS, was not sufficiently efficient or reliable for HTS. Therefore, instead of using the entire S1, we used a 12-amino acid peptide derived from the N-terminal extension of ELC, labeled at its N-terminus with a DABCYL acceptor [28]. This acceptor-labeled N-terminal peptide is designated as ANT. A key advantage of ANT, over our previously used acceptor-labeled myosin [26], is that it can be synthesized and purified reliably in large quantities, thus facilitating large-scale, high-throughput screening (HTS). We hypothesized that compounds affecting the actin-ANT interaction are likely to perturb structural and enzymatic properties of actin-myosin, especially those involving the N-terminal extension of ELC. We measured TR-FRET from actin (labeled at C374 with fluorescein-maleimide) to ANT with the high-precision fluorescence lifetime plate reader (FLTPR) [29] in the presence and absence of compounds from a small-molecule library. This actin-ANT interaction was affected by strong binding of different S1 isoforms and was also perturbed with increasing ionic strength, suggesting overlap between ANT and myosin-binding regions on actin. ANT itself did not have any effect on actin-activated or myofibrillar ATPase. The advantage of using this low-affinity peptide to probe the actomyosin interface was that it was more likely to be susceptible to competition from low-affinity pharmaceutically active compounds and would increase the probability to detect “Hit” compounds. Therefore, FRET between donor actin and acceptor ANT was utilized as a sensor in a high-throughput screening assay to identify compounds that alter the actin-myosin interface. We identified ten “Hit” compounds, from a small (National Clinical Collection-NCC) library, that reproducibly affected FRET with three different preparations of actin and ANT. Those compounds also affected FRET in a concentration-dependent manner, seen in Figure 5. Most of them affected the actin-activated myosin ATPase of both skeletal and cardiac S1 and also altered the structural states of the actin filament. Figure 6 summarizes our findings.

These identified compounds are currently used as medications for various diseases. While these drugs are therapeutically effective, they also have several side effects on muscle function, including cardiac arrhythmia. Thus it is not surprising that these compounds were identified as “Hits”, as they are truly related to the alteration of muscle function. These undesirable side-effects are probably related to our observed changes in actin structural dynamics, which affect the actin-myosin interaction. The effectiveness of a drug depends on the balance between therapeutic benefit and undesired side effects and is probably related to the relative binding affinities to actin and other cellular targets. These “Hit” compounds affect actin when present at µM concentrations, indicating moderate actin binding affinity. If binding to therapeutically desirable targets is much stronger, therapeutic effects may be achieved at low enough doses to avoid effects on actin structure. The drug-induced alteration of actin structure can be also beneficial in cancer research as a method of inhibiting the actin cytoskeleton, which is needed for proliferation of cancer cells. These compounds did not alter the basal Mg-ATPase (without actin) of either skeletal or cardiac S1, confirming their actin-specificity. In addition, FRET concentration-response [28] with two known myosin-specific compounds [47,48] was unaffected, providing further indication of their actin-specificity. This is an exceptional finding, since previous drug discovery campaigns in this field used myosin as the primary target [47,48]. In future screening of larger libraries, we will focus on finding compounds that are specific for skeletal or cardiac muscle. 

## 5. Conclusions

Using the high precision of TR-FRET technology, coupled with structure-based design of an intermolecular FRET biosensor involving actin and the N-terminus of myosin’s ELC, we have established and validated a unique platform for discovering small-molecule effectors of the actin-myosin interaction. This tool can be used in the future to screen larger libraries, and it sets the groundwork for the discovery of allosteric modulators of other actin-binding proteins of interest where protein-protein interactions are targeted.

## Abbreviations

ELC	Essential light chain
RLC	Regulatory light chain
CD	Catalytic domain
LCD	Light chain domain
TR-FRET	Time-resolved Fluorescence resonance energy transfer
HCM	Hypertrophic cardiomyopathy
NCC	National clinical collection
FLTPR	Fluorescence lifetime plate reader
ANT	Acceptor labeled N-terminal peptide
